# MOG-IgG-associated encephalitis and bronchopneumonia in a child with negative infection screening: a case report and literature review

**DOI:** 10.3389/fped.2026.1756701

**Published:** 2026-05-21

**Authors:** Yan Sun, Xin Dong, Mingyue Sun, Fei Wang, Qing-Qing Yu, Yibing Yan, Na Cheng

**Affiliations:** 1 Department of Pediatrics, Jining No. 1 People’s Hospital, Jining, China; 2 Clinical Research Center, Jining No. 1 People’s Hospital, Jining, China; 3 Clinical Medical College (Affiliated Hospital) of Jining Medical University, Jining, China; 4 Department of Gynecology, Jining No .1 People’s Hospital, Jining, China; 5 Department of Ultrasonography, Jining No .1 People’s Hospital, Jining, China; 6 Department of Pediatrics, The First Affiliated Hospital of Shandong First Medical University (Qianfoshan Hospital of Shandong Province), Jinan, China

**Keywords:** bronchopneumonia, child, encephalitis, glycoprotein-associated, myelin, oligodendrocyte

## Abstract

Myelin oligodendrocyte glycoprotein (MOG) antibody-associated disorder (MOGAD) is a central nervous system demyelinating disease in children, frequently induced by infection. We report the case of a 9-year-10-month-old boy with a 13-day fever and headache. He was admitted to our hospital for fever of unknown origin. He was diagnosed with bronchopneumonia via chest X-ray and encephalitis via cranial magnetic resonance imaging (MRI) and laboratory tests, which revealed increased white blood cells (WBC) (67 × 10^6^/L) and a high proportion of segmented granulocytes (31%) in cerebrospinal fluid (CSF). Further examination showed positive serum MOG-immunoglobulin G (IgG). After treatment with high-dose methylprednisolone (20 mg/kg/day, 3 days) and gamma globulin (400 mg/kg/day, 5 days), the patient's temperature returned to normal with decreased CSF WBC count (11 × 10^6^/L) and segmented granulocytes (5%). No abnormal bacterial growth was found in CSF. Oral methylprednisolone (32 mg/day), calcitriol soft capsules (1 capsule/day), and calcium carbonate D3 tablets (1 tablet/day) were prescribed after discharge, with a follow-up examination 1 week later. We present a rare case of a child with fever of unknown origin, diagnosed with MOG-IgG-associated encephalitis, bronchopneumonia, and MOG-IgG seropositivity despite negative infection screening. This report offers new insights into atypical MOGAD and a reference for MOGAD diagnosis and treatment in children. It highlights the importance of conducting MOG-IgG tests in children with encephalitis and bronchopneumonia despite negative infection screening.

## Introduction

Myelin oligodendrocyte glycoprotein (MOG) is a highly conserved glycoprotein uniquely expressed in oligodendrocytes within the central nervous system (CNS). Antibodies against MOG have been recognized for nearly 30 years and are associated with neurological diseases (1). MOG-immunoglobulin G (IgG) antibodies induce various demyelinating syndromes, including optic neuritis, transverse myelitis, acute disseminated encephalomyelitis (ADEM), brainstem syndromes, and cerebral cortical encephalitis (2, 3). Unlike neuromyelitis optica spectrum disorders or multiple sclerosis (MS), MOG antibody-associated disease (MOGAD) is an inflammatory CNS disease with an approximate prevalence of 1.3−2.5/100,000 (4). Notably, MOGAD is more common in pediatric patients than in adults with acquired demyelinating disease (1), accounting for approximately one-third of all demyelinating syndromes in children (5), with ADEM and encephalitis being the most common (6). Infection is thought to be a crucial factor leading to MOGAD. Researchers have reported the case of a 7-year-old girl with MOG-IgG-positive encephalitis after severe acute respiratory syndrome coronavirus 2 infection (7) and a 5-year child with MOGAD presenting ADEM following *Mycoplasma pneumoniae* infection (8). However, there have been no reports of MOG-IgG-positive encephalitis without obvious infections in children.

Herein, we report the case of 9-year-10-month-old boy admitted to our hospital due to a 13-day fever of unknown origin. He was diagnosed with bronchopneumonia, encephalitis, and positive serum MOG-IgG. We aim to expand current knowledge of MOGAD features and provide a reference for the diagnosis and management of atypical pediatric MOGAD.

## Case presentation

A 9-year-10-month-old boy was admitted to our hospital on 28 December 2024, with a 13-day history of fever. He was hospitalized for bloody stools 6 days after birth. He had been rehabilitated for 4 years due to cerebral palsy. At the age of 3, he underwent surgical treatment at a local hospital for hernia. In the past year, he had been treated for depression at the Shandong Provincial Mental Health Center, and he was currently orally taking paliperidone extended-release tablets (alenine), aripiprazole, sertraline, sodium valproate, and benzhexol. He was suspected to have Virchow–Robin spaces and was diagnosed with nasosinusitis on 28 January 2024. Thirteen days prior to admission, the patient developed fever with a maximum temperature of 38℃, accompanied by headache, which was relieved after the fever subsided. He continued to have a fever even after taking oseltamivir phosphate orally for 3 days, and he subsequently received intravenous infusion of ceftriaxone sodium (21 December 2024 to 25 December 2024) and reduning injection (21 December 2024 to 23 December 2024) at a local hospital. Due to persistent fever after treatment, he was given intravenous infusion of cefotiam, reduning injection, and ganciclovir at a nearby clinic for 3 days. On 28 December 2024, laboratory tests revealed the following: white blood cells (WBC) 18.95 × 10^9^/L, neutrophils 80.6%, lymphocytes 14.7%, red blood cells 4.06 × 10^12^/L, hemoglobin 121 g/L, platelets 261 × 10^9^/L, and C-reactive protein 0.29 mg/L. The *Mycoplasma pneumoniae* IgM antibody test was negative. He was treated intravenously with cefotaxime sodium, peramivir, vitamin C, and vitamin B6 at the Shandong Provincial Hospital for 1 day, and then admitted to our hospital for fever of unknown origin. The patient had no allergies and denied trauma, blood transfusion, hepatitis, tuberculosis, and COVID-19. His parents were healthy, and there was no family history of similar symptoms or metabolic diseases. Written informed consent was obtained from the patient's legal guardian.

**Figure 1:**
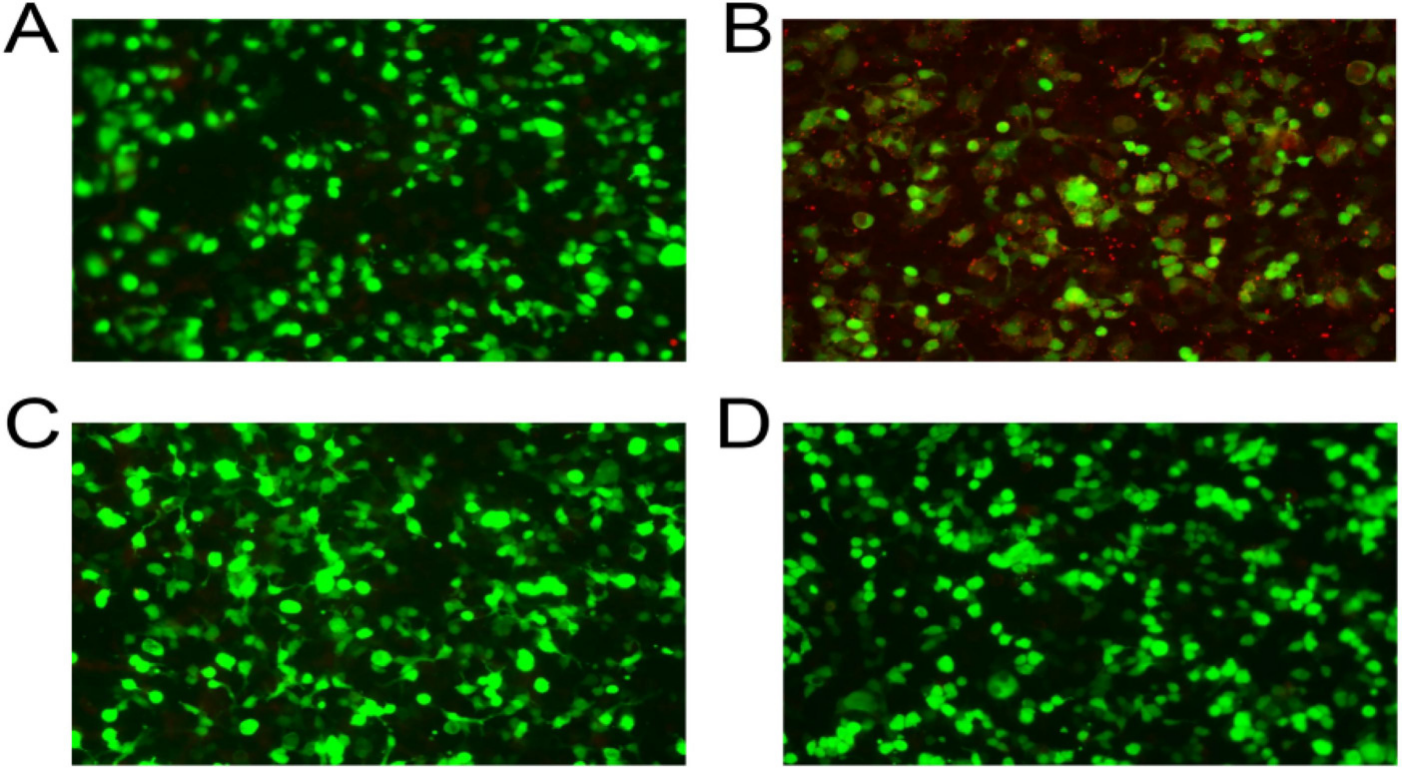
Detection of four central nervous system demyelinating disease autoantibodies in serum. (**A**) Fluorescence intensity of AQP4-IgG in serum. (**B**) Fluorescence intensity of MOG-IgG in serum. (**C**) Fluorescence intensity of MBP-IgG in serum. (**D**) Fluorescence intensity of GFAP-IgG in serum. Four autoantibodies were detected by Nanjing Xiansheng Medical Laboratory Co., LTD, China. AQP4, aquaporin-4; MOG, myelin oligodendrocyte glycoprotein; MBP, myelin basic protein; GFAP, glial fibrillary acidic protein.

On physical examination, the patient exhibited rough breath sounds in his lungs and a 2-cm palpable swollen spleen under his ribs. Other physical examinations including the three-concave sign, Babinski sign, Brudzinski sign, and Kernig sign were negative. Chest X-ray revealed increased lung texture on both sides, with scattered, fuzzy, small patchy shadows visible in the middle and inner bands of the lung fields, which were distributed along the lung texture, suggesting the possibility of bronchopneumonia. Cranial magnetic resonance imaging (MRI) revealed spotty abnormal signals near the posterior horn of the left lateral ventricle, which were considered consistent with enlarged Virchow–Robin spaces. Additional findings included an elliptical cerebrospinal fluid-like signal in the right temporal pole, suggestive of an arachnoid cyst, and a round long T1/long T2 signal in the right sphenoid sinus interpreted as a sphenoid sinus cyst. These MRI findings were regarded as incidental or non-specific and were not considered radiological evidence of active encephalitis, as no definite cortical or subcortical inflammatory lesions were identified. Therefore, the diagnosis of encephalitis in this patient was based primarily on the clinical manifestations and laboratory findings rather than neuroimaging abnormalities alone. General bacterial culture (automated cultivation) results showed viridans streptococci and Neisseria sicca, while blood culture revealed no bacterial growth or fungal growth after 5-day cultivation. A comprehensive workup for infections was negative for Herpes simplex virus IgM, Epstein–Barr virus IgM, D-Dimer, 10 infection markers, Aspergillus, Cytomegalovirus IgM, Rubella virus IgM, and Toxo IgM. Due to the unstable body temperature of the patient, we performed bone marrow aspiration and lumbar puncture for examination on the 6th day of hospitalization. WBC count was 67 × 10^6/L in cerebrospinal fluid (CSF) and the proportion of CSF segmented granulocytes was 31% (Table 1). Based on the examination results, we considered the possibility that the patients had encephalitis. Meanwhile, serum MOG-IgG was positive (1:100+) (Figure 1B), which was the definitive finding confirming MOGAD on hospital day 6. Aquaporin-4 (AQP4)-IgG (Figure 1A), myelin basic protein (MBP)-IgG (Figure 1C), and glial fibrillary acidic protein (GFAP)-IgG were all negative (Figure 1D). Prior to confirmation of MOGAD, differential diagnoses were considered for the patient's prolonged fever, CSF pleocytosis, and pulmonary manifestations, which included the following: (1) infectious encephalitis combined with bacterial bronchopneumonia (ruled out due to negative broad-spectrum infectious screening and no abnormal bacterial growth in CSF after antibiotic treatment); (2) viral meningoencephalitis (excluded based on negative IgM antibodies of common neurotropic viruses such as herpes simplex virus and cytomegalovirus); (3) autoimmune encephalitis other than MOGAD (eliminated by negative AQP4-IgG, MBP-IgG, and GFAP-IgG); and (4) systemic inflammatory disease with CNS and pulmonary involvement (excluded due to no evidence of other systemic involvement).

**Table 1:** Parameters in cerebrospinal fluid, blood, and bone marrow on the 6th day of hospitalization and after treatment.

Specimen	Parameters	Values on day 6 of hospitalization	Values after treatment	Reference range
Cerebrospinal fluid (CSF)	White blood cells ( × 10^^6^/L)	67	11	1–3
	Mononuclear cells (%)	69	95	0
	Segmented granulocytes (%)	31	5	0
	CSF IgG (mg/L)	35.40	97.50	0–34
	CSF albumin (mg/L)	168.00	229.00	0–350
Blood	White blood cells ( × 10^^9^/L)	17.75	13.81	4.3–11.3
	Neutrophils (%)	0.753	0.763	0.31–0.70
	Lymphocytes (%)	0.198	0.165	0.2–0.5
	Neutrophils ( × 10^^9^/L)	13.37	10.54	1.8–6.3
	Lymphocytes ( × 10^^9^/L)	3.51	2.28	1.1–3.2
	Red blood cells ( × 10^^12^/L)	3.82	3.43	4.2–5.7
	Hemoglobin (g/L)	117.00	111	118–156
	Blood platelets (10^^9^/L)	324	222	167–453
	Procalcitonin (ng/mL)	0.053	-	0.1–0.5
	Herpes Simplex Virus antibody IgM	Neg	-	<0.1
	(1–3)-*β*-D-glucan (G-test) (pg/mL)	37.70	-	<151.5
Bone marrow	White blood cells ( × 10^^9^/L)	17.75	-	3.5–9.5
	Red blood cells ( × 10^^12^/L)	3.82	-	4.0–4.5
	Hemoglobin (g/L)	117.00	-	120–140
	Blood platelets ( × 10^^9^/L)	324	-	125–350
	Promyelocytes (%)	3.5	-	0.97–2.17
	Myelocytes (%)	0.5	-	0.31–0.97
	Metamyelocytes (%)	6.0	-	6.52–12.36

After admission, the patient was given cefoperazone, sulbactam, and vidarabine. Based on suspected encephalitis, the treatment was changed to vancomycin (3 January 2025 to 7 January 2025) combined with ceftriaxone (3 January 2025 to 7 January 2025) and acyclovir (2 January 2025 to September 2025). Following a positive MOG-IgG result, a diagnosis of MOGAD was established. The patient received high-dose intravenous methylprednisolone (20 mg/kg/day) for 3 consecutive days and high-dose gamma globulin (400 mg/kg/day) for 5 consecutive days. After this treatment, the patient's temperature returned to normal and his condition gradually improved. Methylprednisolone was administered at 32 mg per day as a single dose in the morning upon waking. Further examination revealed clear and colorless CSF, as well as a negative Pandy's test. The WBC count was 11 × 10^6/L and the proportion of CSF segmented granulocytes was 5%, which was lower than the level before treatment. No abnormal bacterial growth was detected in CSF. CSF IgG level was monitored during in-hospital treatment: The initial CSF IgG level on the 6th day of hospitalization was 35.40 mg/L, and the level increased to 97.50 mg/L after immunotherapy (Table 1); serum MOG-IgG was only detected once for positive confirmation (1:100+) and no quantitative dynamic monitoring was performed during treatment. The WBC count decreased from 17.75 × 10^6^/L to 13.81 × 10^6^/L in blood, approaching the reference range (Table 1).

The patient was discharged on January 17, 2025. Postdischarge medications included oral methylprednisolone at 32 mg/day in the morning upon waking, calcitriol soft capsules at 1 capsule/day, and calcium carbonate D3 tablet at 1 tablet/day. The physicians recommended a follow-up examination 1 week after discharge.

## Discussion

Over the last few years, with the development of a new generation of cell-based assays, autoantibodies to full-length MOG-IgG have been proven to be associated with optic neuritis, transverse myelitis, and ADEM, while being less frequently linked to cerebral cortical encephalitis and brainstem presentations (9, 10). To avoid overdiagnosis and inappropriate treatment, MOG-IgG has been proposed as a core criterion for the diagnosis of MOGAD (10). Jarius and colleagues proposed that MOG-IgG seropositivity detected by cell-based assays using full-length human MOG as the target antigen can be used to diagnose MOG encephalomyelitis (11). Real-world cohort studies of patients with atypical CNS inflammation have revealed that low-positive MOG-IgG is commonly related to a longer interval between disease onset and sampling, with approximately 25% of patients showing low positivity after 6 months when tested with MOG-IgG1 assays (12). These results underscore the importance of paying attention to the optimal detection time for MOG-IgG. López-Chiriboga found that 88% of patients (15/17) had persistent serum-positive MOG-IgG following ADEM, suggesting that longitudinal detection of serum MOG-IgG may help predict disease course (13). Recently, Redenbaugh revealed that assessing the CSF MOG-IgG concentration is helpful for the diagnosis of MOGAD in clinically suspected patients with serum MOG-IgG negativity or low positivity (14). In the present case, seropositivity for MOG-IgG was detected at 1:100 +, with negative AQP4 antibody IgG, MBP antibody IgG, and GFAP antibody IgG, providing the diagnostic evidence of MOGAD in the patient.

Headache, seizures, and fever are the most common clinical symptoms in children with encephalitis (15). Notably, encephalitis is a relatively rare clinical presentation of MOGAD compared with other syndromes in children (16). However, a single-center cohort study claimed that encephalitis is an important initial phenotype in children with MOGAD (17). At the time of initial infection, 24% of encephalitis patients had normal initial MRI results, leading Kim and colleagues to emphasize that MOG antibody testing should be performed in all suspected encephalitis patients, even if MRI results are normal. Furthermore, children with encephalitis are older than children with ADEM (median 8.9 vs. 5.7 years) and are more likely to require intensive care treatment as well as to take steroid treatment later on (15). We reported the case of a 9-year-10-month-old boy with fever for 13 days, accompanied by headache. MRI and laboratory test results indicated the possibility of encephalitis. Therefore, serum MOG-IgG was tested and the positive results indicated MOGAD. Timely initiation of high-dose methylprednisolone and gamma globulin treatment significantly controlled the patient's temperature and decreased WBC. No abnormal bacterial growth was found in CSF. This highlights the necessity of detecting MOG-IgG in patients with MRI-suspected encephalitis or even those with normal initial MRI results. Once diagnosed, timely treatment will improve the prognosis of MOGAD patients.

The combined detection of CSF inflammatory markers and serum MOG-IgG had important supportive value in this case. CSF pleocytosis with an increased granulocyte proportion indicated active CNS inflammation, but these findings were non-specific and could not independently confirm MOG-IgG-associated encephalitis. In contrast, serum MOG-IgG positivity was the key disease-associated biomarker. However, according to current diagnostic criteria, the diagnostic value of MOG-IgG results should be interpreted in conjunction with compatible clinical and radiological features rather than as a standalone gold standard. In addition, the reduction of CSF inflammatory indices after treatment provided useful evidence of inflammatory remission and therapeutic response.

Wegener-Panzer described the clinical and imaging features of 10 children with autoimmune encephalitis and MOG antibodies, reporting median 80 white cell count/μL in CSF and cortical as well as deep gray matter involvement (18). Jarius et al. reported that significant CSF alterations are more pronounced in MOGAD patients with acute spinal cord or brain disease than those with optic neuritis, with elevated CSF white blood cells in 54% of samples (19). In our case, we reported an elevated WBC count and a higher proportion of segmented granulocytes in CSF, which might provide CSF profiles for the diagnosis of MOGAD in children with bronchopneumonia. Notably, extensive infectious screening results were negative, a finding inconsistent with Liu and colleagues, who reported three children with MOG-IgG-associated encephalitis due to *M. pneumoniae* infections, accompanied with elevated CSF mononuclear cells and intracranial lesions (20). Mariotto presented a rare case of a 31-year-old Indian man with positive MOG-Abs and widespread white matter involvement, but with negative extensive infectious screening results, underscoring the atypical features of MOGAD and importance of recognition of this condition (21). Similarly, our patient had negative results on extensive infectious screening, even though he presented with a 13-day fever of unknown origin complicated by bronchopneumonia. The recognition of atypical cases is crucial for the diagnosis of MOGAD, and timely treatment can significantly improve MOGAD patient outcomes.

Corticosteroids and adjunct therapies, including intravenous immunoglobulin and plasma exchange, are the standard first-line approaches for acute MOGAD in clinical practice. High-dose intravenous methylprednisolone and gamma globulin treatments have demonstrated benefit in disease control among children with MOGAD, although seven out of 24 children experience a relapse (22). Similar to the cases reported by Wang et al. (22), our patient responded well to high-dose methylprednisolone, but still lacked a long-term follow-up to determine whether it will relapse. Nosadini observed that immunotherapy within 7 days reduced recurrence rates 6.7-fold, corticosteroid therapy lasting more than 5 weeks reduced recurrence rates 6.7-fold, and MRI examination of optic nerve abnormalities reduced recurrence rates 12.5-fold, highlighting the importance of early immunotherapy and longer corticosteroid treatment in reducing the risk of relapse (23). Chwalisz concluded that long-term oral corticosteroid tapers may decrease the risk of rapid relapse in MOGAD (24). In our case, after intravenous high-dose methylprednisolone (20 mg/kg/day) for 3 consecutive days and high-dose gamma globulin (400 mg/kg/day) for 5 consecutive days, the patient achieved disease control. Upon discharge, he was prescribed oral methylprednisolone at 32 mg/day in the morning upon waking, calcitriol soft capsules at 1 capsule/day, and calcium carbonate D3 tablet at 1 tablet/day. Early immunotherapy and longer corticosteroid treatment may be beneficial for children with MOG-IgG-associated encephalitis presenting with prolonged fever of unknown origin and bronchopneumonia. A follow-up examination 1 week after discharge is recommended.

Several limitations should be noted. The patient was discharged on 17 January 2025, and the duration of oral methylprednisolone remains relatively short; therefore, a longer follow-up is needed to determine whether relapse of MOGAD will occur. The patient is currently receiving oral paliperidone extended-release tablets (alenine), aripiprazole, sertraline, sodium valproate, and benzhexol for depression. It is unclear whether these drugs affect MOGAD or increase the risk of relapse. For example, paliperidone and aripiprazole are atypical antipsychotics, which can modulate the body's immune response by regulating cytokine secretion and immune cell activity (e.g., inhibiting the overactivation of T cells/B cells); sertraline is a selective serotonin reuptake inhibitor, which may affect the blood–brain barrier's permeability. However, the specific role of these psychotropic drugs in the occurrence of MOGAD is still unclear, and it is impossible to rule out whether they alter the clinical manifestations of MOGAD (e.g., attenuating neurological symptoms) and affect the diagnostic interpretation. Moreover, the correlation between cerebral palsy and MOGAD remains unclarified. Cerebral palsy is a chronic CNS developmental disorder, which may lead to subtle structural and functional abnormalities of the CNS. Although there is no direct evidence that cerebral palsy is a risk factor for MOGAD, chronic CNS structural changes may increase the susceptibility to autoimmune demyelination, providing a pathological basis for its occurrence. In addition, we did not monitor the serum MOG-IgG concentration after the patient achieved disease control.

In conclusion, we reported the case of a 9-year-10-month-old boy suffering from a 13-day fever of unknown origin, diagnosed with bronchopneumonia and MOG-IgG-associated encephalitis. The patient's body temperature returned to normal and WBC count in CSF was significantly decreased after high-dose methylprednisolone and gamma globulin treatment. This case highlights the importance of recognizing atypical MOGAD in children.

## Data Availability

The original contributions presented in the study are included in the article/Supplementary Material, further inquiries can be directed to the corresponding authors.
